# Video Voiding Device for Diagnosing Lower Urinary Tract Dysfunction in Men

**DOI:** 10.1007/s40846-017-0283-8

**Published:** 2017-06-22

**Authors:** Mehdi Shokoueinejad, Rayan Alkashgari, Hisham A. Mosli, Nazeeh Alothmany, Jacob M. Levin, John G. Webster

**Affiliations:** 1 Department of Biomedical Engineering, University of Wisconsin-Madison, Madison, WI 53706 USA; 2 Department of Electrical and Computer Engineering, King Abdulaziz University, PO Box 80204, Jeddah, 21589 Saudi Arabia; 3 Department of Urology, King Abdulaziz University, PO Box 80204, Jeddah, 21589 Saudi Arabia; 4 6205 Mineral Point Road, Apt 222, Madison, WI 53705 USA

**Keywords:** Urine Flow, Video Processing, Urology, Voiding, Diagnosis, LUTD

## Abstract

We introduce a novel diagnostic Visual Voiding Device (VVD), which has the ability to visually document urinary voiding events and calculate key voiding parameters such as instantaneous flow rate. The observation of the urinary voiding process along with the instantaneous flow rate can be used to diagnose symptoms of Lower Urinary Tract Dysfunction (LUTD) and improve evaluation of LUTD treatments by providing subsequent follow-up documentations of voiding events after treatments. The VVD enables a patient to have a urinary voiding event in privacy while a urologist monitors, processes, and documents the event from a distance. The VVD consists of two orthogonal cameras which are used to visualize urine leakage from the urethral meatus, urine stream trajectory, and its break-up into droplets. A third, lower back camera monitors a funnel topped cylinder where urine accumulates that contains a floater for accurate readings regardless of the urine color. Software then processes the change in level of accumulating urine in the cylinder and the visual flow properties to calculate urological parameters. Video playback allows for reexamination of the voiding process. The proposed device was tested by integrating a mass flowmeter into the setup and simultaneously measuring the instantaneous flow rate of a predetermined voided volume in order to verify the accuracy of VVD compared to the mass flowmeter. The VVD and mass flowmeter were found to have an accuracy of ±2 and ±3% relative to full scale, respectively. A VVD clinical trial was conducted on 16 healthy male volunteers ages 23–65.

## Introduction

In human physiology there are two phases of the micturition cycle: the storage phase and the voiding phase. However, a third post-voiding phase appears with pathological disorders such as Benign Prostatic Hyperplasia (BPH). Thus disturbances of the storage phase are described as storage symptoms and those of the voiding phase are described as voiding symptoms. Voiding symptoms involve abnormalities of the urinary stream such as weakness, loss of force, diminished caliber, intermittency, spraying and deviation of the stream to one side, and straining to void. Post-voiding symptoms are described as post-voiding dribbling and sensation of incomplete bladder emptying.

During the assessment of Lower urinary tract symptoms (LUTS), the clinician obtains a full voiding history, details of the patient’s complaints, and depends heavily on the patient’s narrative description of his voiding disturbances. While storage symptoms are usually due to bladder dysfunction, voiding symptoms are thought to be due to obstruction of the urinary passage by lesions such as BPH, urethral stricture, meatal stenosis and other urethral disorders such as stones, malignancy or traumatic lesions. A major and essential step in the medical evaluation of LUTS is physical examination of the patient. This usually focuses on the examination of the urinary bladder and the prostate gland. However, there have never been any published documents on the actual observation of terminal or post-void dribbling and no direct examination of elements of voiding symptoms by watching the urinary stream during voiding and observation of the patient’s facial expression during straining. Therefore, the clinician judges the severity of these voiding symptoms depending only the description given by the patient without actual verification or documentation.

This paper describes a novel method to examine the voiding and post-voiding phases of micturition for voiding symptoms. The method enables a physician to verify a patient’s description, and evaluate any voiding and post-voiding symptoms by visually documenting the whole voiding process and measuring any urological parameters, such as flow rate, simultaneously. Therefore it is an improvement to the existing diagnostic tools, and would help greatly in the follow-up assessment of patients.

Video and image processing are essential parts of modern medical image analysis and computer vision-aided healthcare services. Urinary voiding visual inspection plays a useful role in the diagnosis of a number of urological disorders. When an expert eye is allowed to visualize and examine a disorder, a solid conclusion can be made and a therapeutic decision is based on clear evidence, the power of vision. Traditional testing of voiding such as uroflowmetry, urethrography and urethral calibration do not include an actual visual examination of voiding by the clinician’s eye [1]. They produce graphic traces, radiological images and impressions that are less helpful than watching the act itself [2]. Furthermore, the confirmation of clinical diagnosis of some voiding disorders such as stress urinary incontinence depends solely on visual inspection. For instance, visualization of the urine leakage from the urethral meatus with a cough is a diagnosis of stress urinary incontinence [3].

Most patients are willing to allow their urologists to observe them during voiding or at times of urinary incontinence in order for the treating urologist to make a judgment of the condition that would help in finding the correct solution for the problem. Patients lack the expertise to estimate and describe the subjective impression of the force and caliber of their urine flow since they do not have a means to compare with others; some of them just don’t observe their urine flow by nature such as men who are not able to make this observation because of their shape, weight, age, or illness [4]. Documentation and post-processing of the video footage of the urination provide useful information for the urologist to diagnose the urinary problems.

Lower urinary tract dysfunction (LUTD) is common in both men and women, and the incidence and prevalence increase with advancing age [5]. Symptoms of LUTD encompass all urinary symptoms including storage, voiding, incontinence, and post-micturition symptoms [6]. “Symptoms of LUTD are highly prevalent and occur in both genders to a similar extent, with 51% of men and 59% of women exhibiting storage symptoms; 26% of men and 20% of women exhibiting voiding symptoms; and 17% of men and 14% of women exhibiting post-micturition symptoms. The impact and burden of symptoms of LUTD to individuals and to the nation are enormous. Those patients with symptoms of LUTD suffer considerable morbidity resulting in a significant decrease in quality of life for both the patient and his/her partner.” [7].

The VVD visually documents urinary voiding events remotely. In addition, it also has the ability to measure the instantaneous flow rate at any given moment in time during urinary voiding. The VVD should improve the initial diagnosis of LUTD, and also improve the evaluation of LUTD treatments by providing subsequent follow-up documentations of voiding events after treatments. The VVD enables a patient to have a urinary voiding event in privacy while a urologist monitors, processes, and documents the event from a distance. By helping to make the correct diagnosis and evaluating the degree of LUTD severity, the VVD should be of clinical importance, especially in the documentation and further monitoring of the progress of the LUTD condition.

The following should be the clinical application of the use of this device that reflects its importance in the diagnosis and management of LUTD, therefore helping to improve this condition:

1. In the initial confirmation of the presence of Lower Urinary Tract Symptoms (LUTS), as claimed by the patient (diagnosis).
2. Documentation of the type and degree of urinary incontinence (UI): such as urine leakage during physical stress. The degree of stress urinary incontinence (SUI) is determined by direct measuring of the actual amount of urine leaked.
3. In the follow-up monitoring of the various types of treatment such as:
  - Physiotherapy and pelvic floor exercises: the VVD should help in monitoring the progress of UI following this type of therapy.
  - Medical treatment of UI: the VVD should help in monitoring the effectiveness of medical treatment of diseases such as Benign Prostatic Hyperplasia (BPH) and bladder neck obstruction.
  - Surgical treatment of UI: the VVD should help in monitoring the success or failure of surgical management, the degree of improvement of UI can be assessed and documented following this type of therapy.

This study has two primary objectives. The first is to document the urine voiding process by video recording the urine stream from two angles (top and side). Observing the shape of the urine stream can be a practical diagnostic tool for medical practitioners since it provides a noninvasive method of estimating subjective impression of the force and urethral dilation. The second objective is to extract parameters that would benefit the urologist in making better diagnoses and decisions, such as the Maximum (Peak) Flow Rate, Average Flow Rate, Instantaneous Flow Rate, Total Voiding Time, and Total Voided Volume. We have analyzed and measured the important urological parameters with VVD using the proposed video processing algorithm.

The rest of the paper is organized as follows. Section 2 examines the related work and the background. Section 3 describes the overall methodology. Results and methodology validations are discussed in Sect. 4 and its different subsections. Finally, discussion, conclusion and future directions of the present work are discussed in Sects. 5 and 6 respectively.

## Background

The devices currently used to study urinary voiding actually measure urine-flow or volume per unit time (also called uro-flow-rate), time of voiding, average flow rate over the total voiding time etc. [8]. Among those, the most important parameter is the maximum flow rate (*Q*-max.) or the peak flow rate defined as the highest point on the curve the flow reaches during the period of voiding [9]. This is purely physics and recorded by a device that depends only on the physical properties of the voided urine, that is weight and the speed of its accumulation [10]. It records the measurement at the end of the urine stream or column at a point the stream may have already been slowed, branched, bifurcated or turned into a spray rather than one solid stream. Therefore, other important clinical characteristics of the act of voiding and the nature of the voided urine are not observed. Clearly, to the urologist, learning about the subjective impression of the urine force during its natural flow as visually observed and noting its direction and caliber is equally important especially in certain medical circumstances such as obstructed flow of urine, deviated stream [11], stress urinary incontinence and conditions associated with interrupted stream such as detrusor sphincter dyssenergia [12].

Researchers have suggested simple devices that can measure flow in the home or office [13–16]. Today’s technology allows us to use miniature devices to provide a clear view for the treating urologist to make the needed diagnosis. This device can be used for men, women and children at different age groups. It is more convenient for both the patient and the urologist to observe the act of voiding remotely using a monitor while the patient is standing or seated comfortably in separated privacy.

One of the simple approaches, namely visual examination of the urinary stream without taking any measurements has also been recommended for assessing voiding ability in children [17]. This approach provides an estimation of the strength of the stream; it can scarcely be viewed as quantitative.

Another method to assess voiding ability used 8 mm film to measure velocity of the urinary stream [18]. This method experienced some of the more common drawbacks inherent in other routines.

Photography of the urinary stream was later utilized and showed that patients’ urinary stream breaks into drops when it leaves the external meatus [19]. By using light-beam interruption methods they measured the volume, and velocity patterns of the drops. Nonetheless this creative technique was used as a tool for urological research and it was used to obtain voiding patterns of normal and obstructed males but it had drawbacks that the rendered result was unsuitable for routine clinical and office use [20]. It did not provide quick outcome, cost and complexity were high, and expertise was required for the translation of the results.

A picture taken in 1906 showed a boy urinating following surgical correction of a congenital urethral anomaly [11]. At that time no urodynamic machine was developed yet and the visual inspection of the still picture served the purpose of demonstrating the good urinary stream just fine.

Researchers advocated a “new” way to help patients to describe their symptoms and encompass a picture (silhouette) of a urinating man with different flow magnitudes in order for the patient to compare his own stream with one of them [1]. Video recording of the patient’s stream live and inspecting it by the urologist is much more realistic, practical and convenient especially if it can be sent by e-mail over the internet to the urologist in his office.

Therefore, the need is evident to find a better way to examine the urine flow during the act of voiding to note its subjective impression direction, force and caliber. By studying previous work, the VVD is capable of monitoring the urine stream with three cameras, which helps the urologist to learn about the force and direction of the urine during its natural flow.

The VVD and its method can be used alone or in conjunction with the traditional urodynamic study devices. This should be an optimal combination to study the act of voiding from all its aspects. VVD can improve evaluation of LUTD treatments by providing subsequent follow-up.

## Materials and Methods

This section describes the overall design and the proposed video processing algorithm and software.

### Description of the Overall Experimental Set Up

**Fig. 1:**
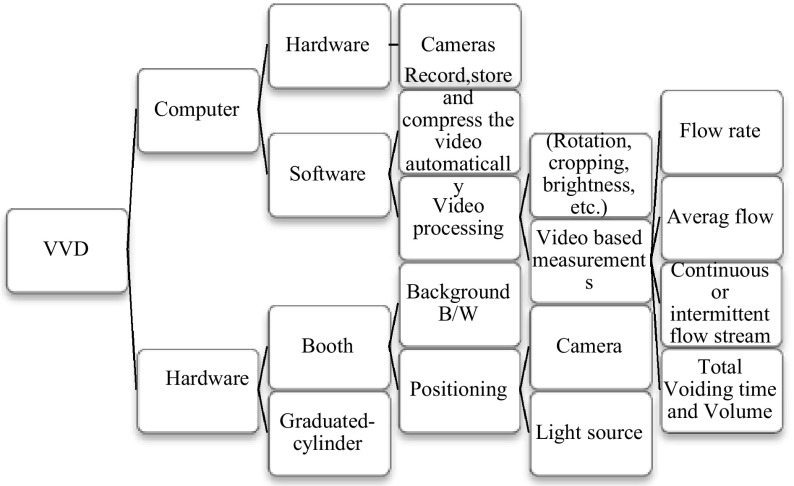
VVD development requires (*top*) computer hardware and software, cameras, video processing, storage and transmission and (*bottom*) hardware for the booth, toilet, and graduated cylinder [The part where the patient actually voids (urinates) into]

The VVD consists of a lightweight plastic booth with LED lights, and video cameras on the left, top, and lower back sides. The left and top cameras are used to visualize urine leakage from the urethral meatus, urine stream trajectory, its break-up into droplets, etc. The lower back camera monitors a cylinder where urine accumulates. Software then processes the change in level of accumulating urine in the cylinder and calculates parameters like the Maximum (Peak) Flow Rate, Average Flow Rate, and Instantaneous Flow Rate. Figure 1 shows an overview map of the VVD components divided into hardware and software.

The VVD has three positioned cameras. The side camera views the urine stream from a 90° horizontal angle on a black background. The top camera views the urine stream from a 90° vertical angle on a black background. The cameras used here were a Logitech c920, which is a digital full HD 1080p pro Webcam, with 20-step autofocus lens. The lower back camera (the cylinder camera) monitors the accumulation of urine into a 1000 ml cylinder. Three G-LUX series 8 W LED Spot Lights illuminate the stream to provide best contrast.

Two LEDs are mounted on the wall facing the patient (above and below the urine stream line of action). One LED is mounted at the top of the right side wall, shedding light from a steep downward orientation/trajectory towards the funnel. The combination of these three LED placements shedding light from three different angles results in complete illumination of the urine stream, ensuring high contrast and visibility.

**Fig. 2:**
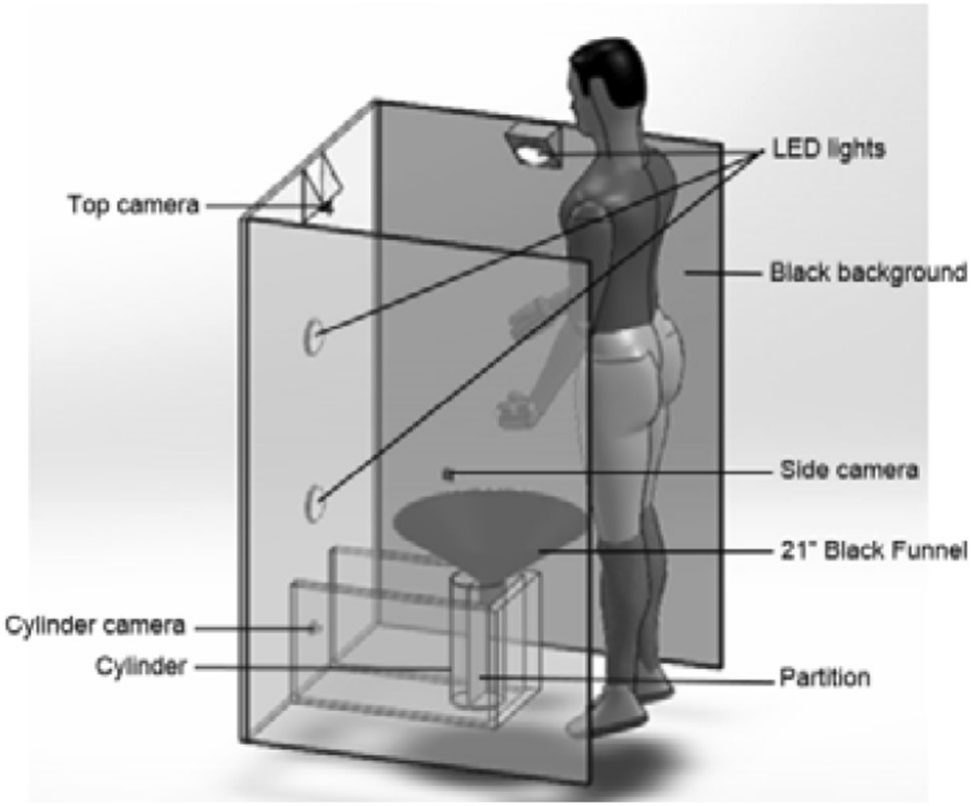
A perspective view of one VVD booth setup illustrating a male patient standing ready to urinate with three cameras monitoring from the *side*, *top*, and *back* (*cylinder view*)

To use the VVD, the patient stands in front of the device and urinates. The urine is guided by the large 53 cm diameter funnel leading it to the cylinder. The urination is captured from two angles using two cameras (top and side). The cylinder camera captures the filling process of the cylinder in order to compute the instantaneous flow rate, max flow rate, total voiding time, and total voided volume. (See Fig. 2).

### Description of the Developed Software

Software was developed using MATLAB to analyze the video recording of the urine accumulating in the cylinder and calculate the instantaneous flow rate, max flow rate, total voiding time, and total voided volume. However, urine can sometimes be clear in color, which makes it very challenging to be traced using a camera. So a blue hollow piece of plastic (floater), which can be recognized by our software, is added at the bottom of the cylinder prior to the voiding process. As urine flows in, the floater floats on the urine surface and rises to keep track of urine level in the cylinder. To help avoid water start-up artifact and any errors in measurement that may arise due to non-uniform graduate cylinder bottoms, the graduate cylinder is prefilled with a predetermined amount of water enough to float the floater [21].

**Fig. 3:**
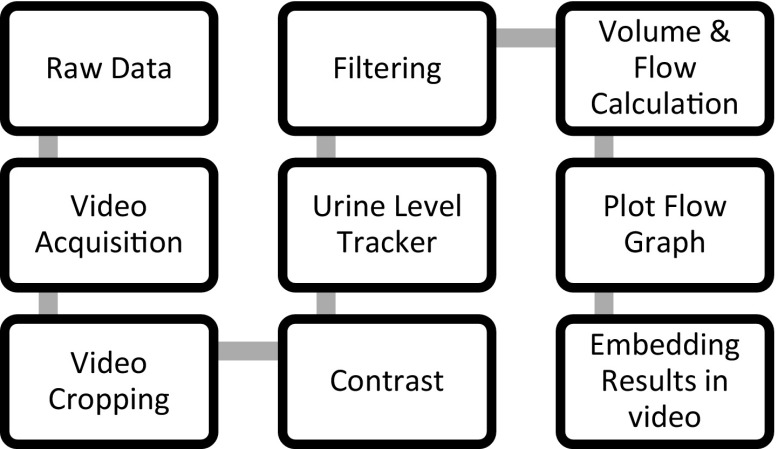
The software acquires the video, processes it, and displays images and flow versus time

Figure 3 shows the software starts by acquiring the video frames and isolating the cylinder from its surroundings. Each frame is then cropped to only show the middle part of the cylinder. Contrast is enhanced by binarizing the image. After that an algorithm tracks the level of urine in the cylinder and measures the volume at each frame. A series of moving average filters (ranging from 21-term to 3-term moving average) are applied on the graph of volume versus time to smooth it as much as possible without deforming it. As a result, differentiating the volume versus time gives a smooth and precise flow graph without any noisy fluctuations. Both the flow graph and instant flow at each frame are embedded within the video to provide a deeper insight by allowing the urologist to track the flow instantaneously as the patient urinates.

## Results

Performance of the proposed VVD was evaluated by recording and processing 21 bench-testing videos of simulated voiding at the instrumentation laboratory of University of Wisconsin-Madison (described in Sect. 4.1) and 3 clinical-testing videos on volunteer patients at department of urology of King Abdulaziz University, (described in Sect. 4.3).

### Method Validation (Bench Testing)

Method validation is the process that we used to confirm that the analytical and computational procedure employed for our video processing is suitable. Results from method validation were used to judge the quality, reliability and consistency of analytical results.

**Fig. 4:**
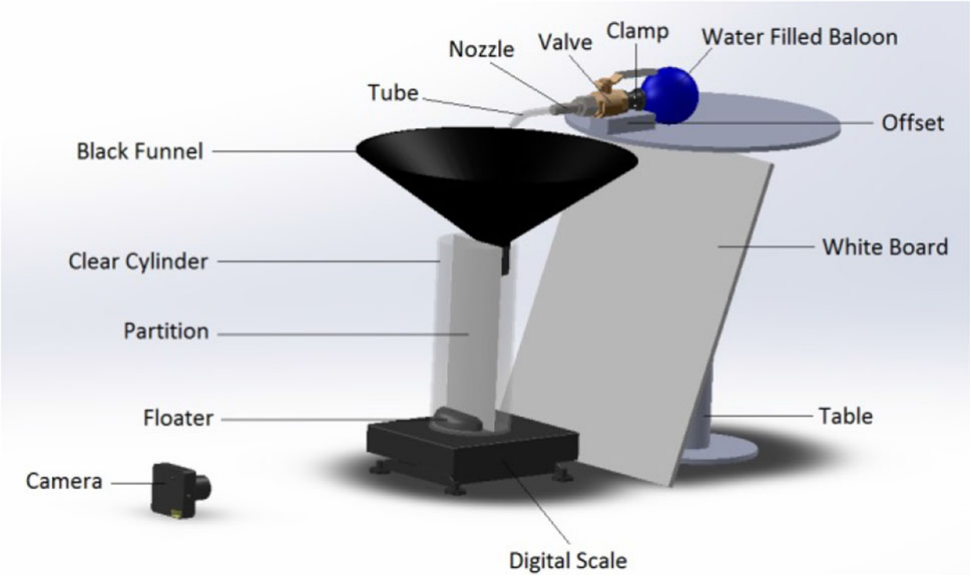
Bench testing setup—A balloon squeezes out a water stream directed by a tube toward a black funnel that guides the stream into a graduated cylinder sitting on a digital scale. The camera monitors the graduated cylinder and records volume versus time, which is used to derive flow versus time. The digital scale (iWeigh-002 M-6D-DI100-LV1000 model by Loadstar Sensors) records weight versus time, which is used to derive flow versus time

In order to validate the precision and accuracy of the flow rate measurements based on the video processing approach VVD, a scale was used as a mass flowmeter in an experiment to compare its flow rate measurements to that of the VVD. The experiment was designed so that it simulates human urinary voiding. A water filled balloon was used to simulate the function of a human bladder. As the balloon wall puts pressure on the water inside it, it squeezes out the water through a tube that simulates the urethra. The tube directs the water toward a funnel resting on top of a graduated cylinder and a scale. Data from the VVD camera and mass flowmeter scale are acquired simultaneously (see Fig. 4).

**Fig. 5:**
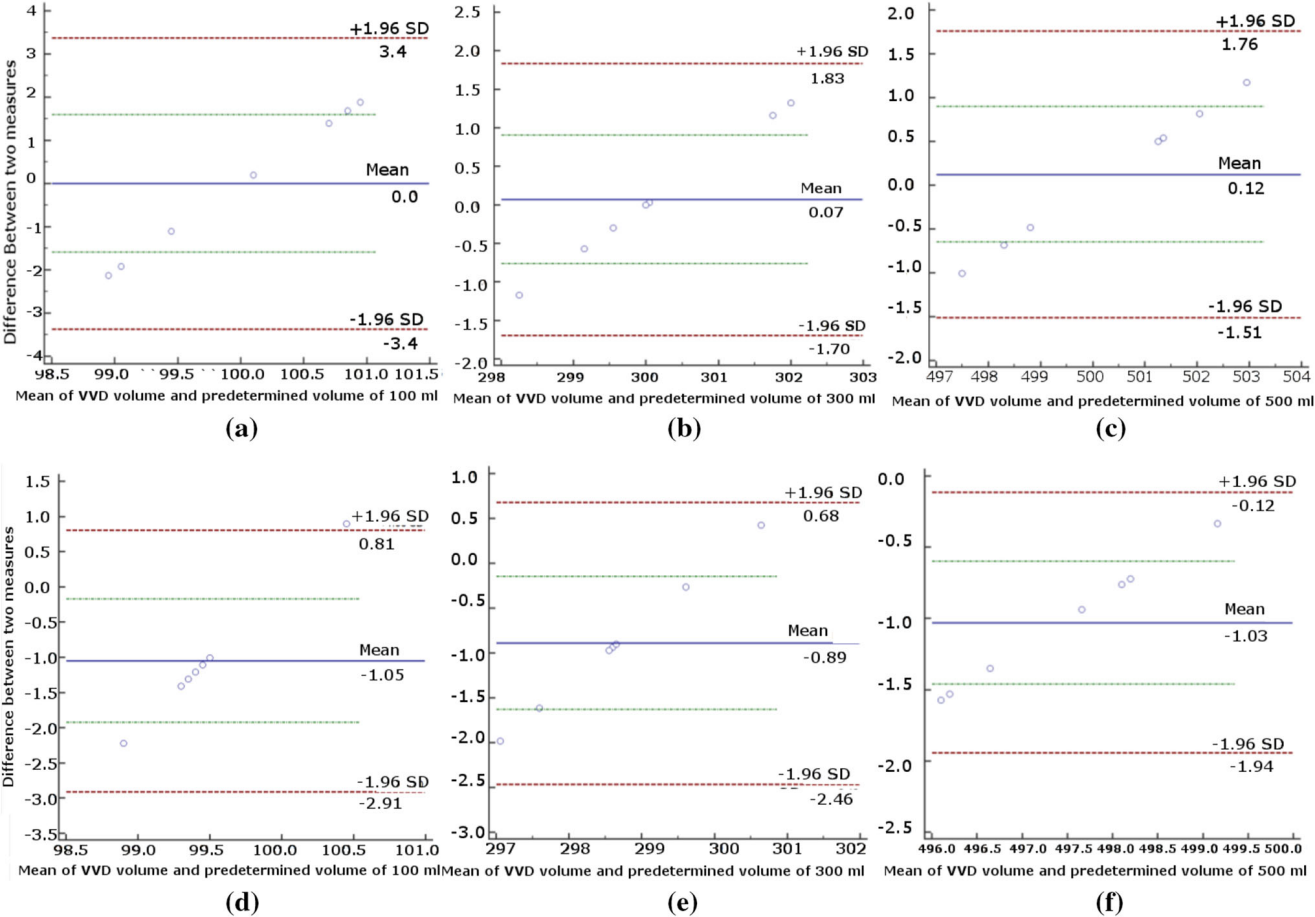
**a–c** A Bland–Altman plot showing the difference between the total voided volume measured by the VVD and the true total voided volume of **a** 100 ml **b** 300 ml **c** 500 ml. **d–f** A Bland–Altman plot showing the difference between the total voided volume measured by the mass flowmeter and the true total voided volume of **d** 100 ml. **e** 300 ml **f** 500 ml. The *green lines* represent the 95% confidence interval

**Fig. 6:**
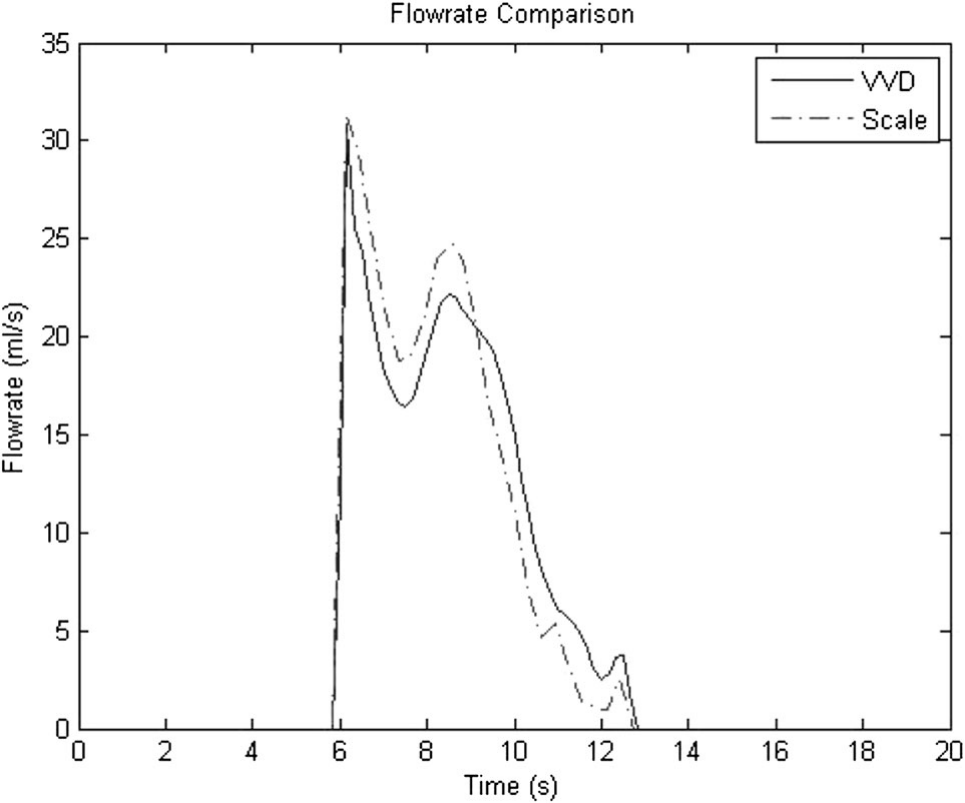
Flow rate versus time as measured by the VVD and scale mass flowmeter

The validation experiment was done using 3 accurately measured predetermined volumes of water as a gold standard (100, 300, and 500 ml). To give a reasonable assessment of accuracy and repeatability, for each volume, the validation experiment was repeated 7 times. A method of data plotting, Bland–Altman plot, was used in analyzing the agreement between two different assays for VVD vs. predetermined volumes and mass flowmeter vs. predetermined volumes, respectively (see Figs. 5, 6) [22]. Figure 5a–c show the mean differences between the predetermined total voided volumes (100, 300, and 500 ml) and the total voided volumes measured by the VVD, a new technique, are 0, 0.07, and 0.12%, respectively. A positive mean difference indicates that the VVD tends to give a little bit higher reading of the 95% confidence interval. Figure 5d–f show the mean differences between the predetermined total voided volumes (100, 300, and 500 ml).The total voided volumes measured by the mass flowmeter for these three instances are −1.05, −0.89, and −1.03%, respectively. A negative mean difference indicates that the mass flowmeter tends to give a little bit lower reading of the 95% confidence interval. However, the fact that at least 95% of the differences, for the VVD and mass flowmeter, are less than two standard deviations and the mean differences are close to zero indicates both systems are repeatable and accurate [22]. Table 1 shows the VVD and mass flowmeter both produce accurate repeatable results.

**Table 1:** Comparison between VVD and mass flowmeter measurements of predetermined voided volume−100*(mean ± SD)/predetermined volume (%)

Predetermined voided volumes (ml)	Voided volume accuracy (VVD)	Voided volume accuracy-mass flowmeter
100	100 ± 1.6	99 ± 0.9
300	100.1 ± 0.9	99.1 ± 0.8
500	100.1 ± 0.8	99 ± 0.5

Figure 6 compares flow rate versus time as measured by the VVD and mass flowmeter. It shows that the VVD is comparable to the mass flowmeter, and is able to detect the start and ending points of the voiding period. The maximum flow rate and the overall shape of the voiding periods are relatively close together. As the urine stream descends through the funnel and graduated cylinder, a delay occurs between the start of voiding and the start of flow rate measurements. However, this delay presents itself as a shift in time of the whole voiding flow graph and so it can be shifted in time to synchronize with the start of voiding.

### Measuring VVD Frequency Response

The step response test is used to determine the frequency response of an uroflowmeter. A steady flow using the bottle described in Griffiths et al. paper was used to measure the frequency response of the first-order system [16]. Due to assumption of a first order system with one time constant *τ* the measured flow will drop to 37% of its starting value [14]. By using the logarithmic method given in (1) and (2) the calculated cut off frequency of the VVD is equal to 5.5 Hz.

1 $$\tau = \frac{\Delta t}{{\Delta \log_{e} Q}}$$

2 $$f_{\text{c}} = \frac{1}{2\pi \tau }$$

where *Q* is the flow rate, *t* is time, and *f*_c_ is cut off frequency.

### Clinical Testing

**Fig. 7:**
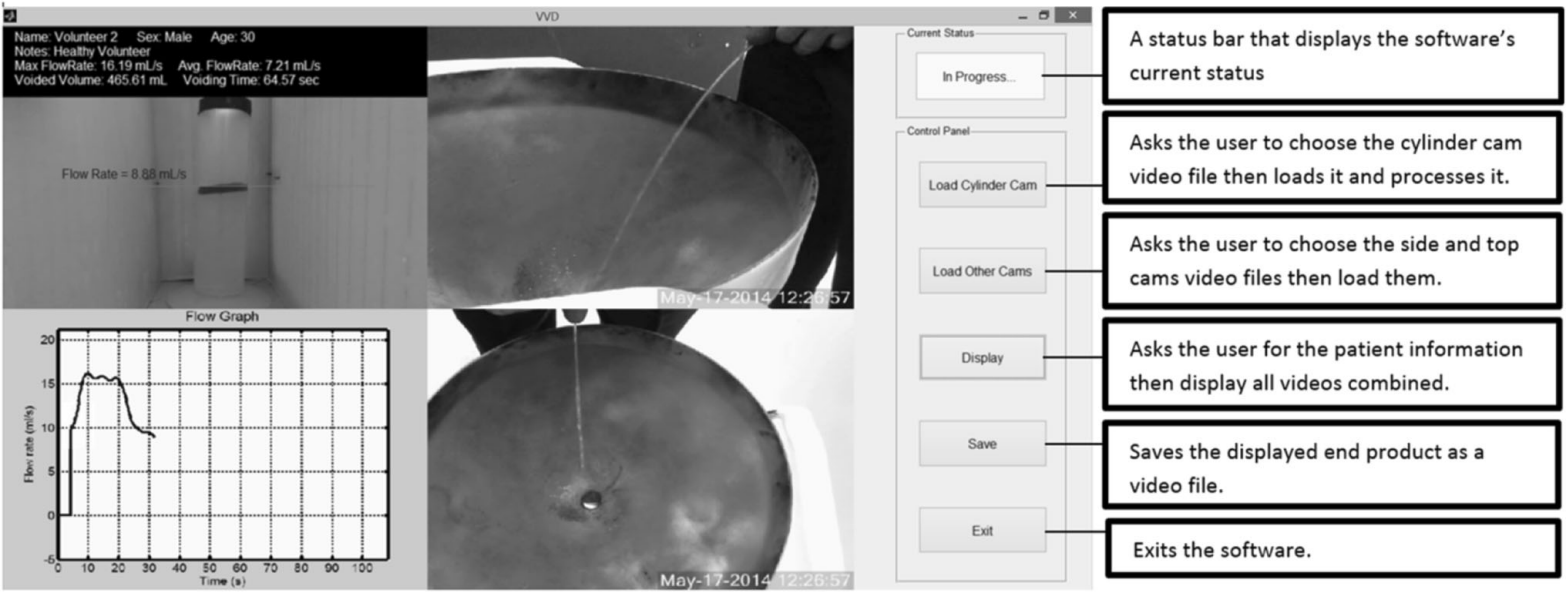
Screenshot of the VVD software mid-voiding displays all three cameras, patient information, uroflowmetry results, and a live flow graph

Clinical testing was done on 3 male subjects (age 46–65). The feedback from human testing helped significantly in further development and fine tuning of the VVD software. Table 2 shows the results obtained by the VVD software from three recording sessions of three healthy volunteers. The average flow rate was found to be 12.7 ml/s. Similarly, the US National Library of Medicine (2014) states that the average flow rate in males between the ages of 46–65 is 12 ml/s [23]. Figure 7 shows the VVD software displaying all three cameras plus a live flow graph mid-voiding and post-voiding, respectively. The software also displays the patient’s name, sex, age, case notes, instantaneous flow rate, max flow rate, average flow rate, voiding time, and voided volume.

**Table 2:** Clinical testing measurements of 3 healthy volunteers

Volunteer no.	Max. flow rate (ml/s)	Average flow rate (ml/s)	Voiding time (s)	Voided volume (ml)
1	24.4	16.1	21.9	352.6
2	16.2	7.2	64.6	465.6
3	20.0	14.9	44.8	667.5

## Discussion

The International Continence Society (ICS) technical report recommends the following standards: a range of 0–50 ml/s for *Q*_max_, a range of 0–1000 ml for voided volume, maximum time constant of 0.75 s, and an accuracy of ±5% relative to full scale [9]. The VVD is capable of measuring a range of 0–50 ml/s for *Q*_max_, has the capacity to accommodate 1000 ml of voided volume, has a maximum time constant of 0.18 s, and an accuracy of ±2% relative to full scale, thus, satisfying the ICS technical report standards.

The mass flowmeter is capable of measuring a range of 0–50 ml/s for *Q*_max_, has the capacity to accommodate 1000 ml of voided volume, has a maximum time constant of 0.21 s, and an accuracy of ±3% relative to full scale. However, for most mass flowmeters, the fluid content and concentration can induce variations in the fluid specific gravity, which directly influence the measured flow rate. For example, urine of high concentration may increase apparent flow rate by 6% [9]. This would lead to an accuracy of 6% relative to full scale, which does not meet the ICS technical report standards. The VVD is not a mass flowmeter, thus, it is not affected by the fluid specific gravity, which makes it a more accurate source of diagnostic information than existing commercial mass flowmeters.

A urologist has tested the VVD and found the following. The main function of the device is unique, has several important clinical values that have not been described before in any other currently available devices. The Video-based Visual Voiding Device (VVD) is expected to simultaneously perform the following:

1. Defining the subjective impression *direction* of the urine flow in relation to the vertical axis of the human body i.e., deviation to the right or left and in relation to the direction of the flaccid penis i.e., upwards and downwards and determination of the degree of the angle of deviation i.e., 15, 20, 30 up to 90° deviation.
2. Recognition of fluid spraying, splitting and *branching* (bifurcation/trifurcation) of the urine stream.
3. Measure/calculate the *total voiding time* in seconds accurately. The voided urine is collected and its volume is measured. The maximum voiding flow rate and the *average voiding flow rate* (total urine volume divided by total voiding time in seconds) are calculated [24].
4. Video recording, documentation and playback of the whole urinary voiding event from two distinct angles.
5. Video recording, documentation and playback of momentarily loss (leak) of urine during coughing, sneezing and laughing, a condition known as *stress urinary incontinence* in males.

Most of the available household and clinical uroflowmeters do not have the ability to record the video of the voiding process for further diagnosis and analysis, which means the voiding test result of these types of devices is only available in the form of charts and graphs. Also, simple visual examination by an expert can give important qualitative information during patient voiding but this process is not recorded for another colleague’s review and it is hard for them to note the direction, caliber, etc. of the urine stream. The VVD represents a paradigm shift by integrating quantitative urological parameters with recorded qualitative visual examination, and displays all information in real time on one screen for the expert. (See Table 2).

Table 3 shows the primary differentiation factors of VVD with the previous devices, compares the difference between commercial uroflowmeters, simple visual examination, and the VVD. Commercially available flowmeters, which have generally acceptable accuracy, include the following: (1) Weight sensor flowmeter which weighs the urine voided—measuring the volume of urine voided and hence measuring the urine flow rate by differentiation with respect to time. (2) Ordinary pressure sensor in which the hydrostatic pressure exerted by a column of urine also can be applied to measure the weight of the urine voided, and (3) Rotating disc flowmeter which has a spinning disc on which urine falls. Although all these available uroflowmeters can measure some urologic parameters accurately, they cannot record other important clinical characteristics of the act of voiding, such as noting the direction of the urine, the urine leakage, and caliber of the urine. Obviously, in certain medical conditions such as obstructed flow of urine and deviated stream, visually observing and studying aformentioned parameters can provide important information for medical diagnosis [11].

**Table 3:** Comparison of the Uroflowmeters, simple visual examination, and VVD‘s features

	Commercial uroflowmeters	Visual examination	VVD
Observations	Not accessible	Visual, seen by expert eyes one time	Visual, seen by expert eyes, recorded video footage for further analysis
Accuracy	Generally accurate but varies based on device	Estimation only	Accurate (±2%)
Volume and Stream caliber	Volume is measured in ml; stream caliber cannot be observed	Volume cannot be measured; Caliber is observed by urologist (one time)	Volume is measured in ml; Caliber is observed and recorded
Flow rate and direction	Flow is measured in ml per second; direction cannot be measured	Flow cannot be measured; direction can be observed (one time)	Flow is measured as ml per second; direction is observed and recorded
Urine stream continuity	Charted on graph	Observed by urologist (one time)	Charted on graph while being observed by urologist
Observation angle	Not accessible	Observed by urologist from one angle (one time)	Observed by urologist on the screen from two angles and recorded by VVD for future examination

## Conclusions

The VVD uses lower cost components than existing commercial flowmeters. The estimated total parts cost of the VVD in 2015 is $300 which mainly consists of the 3 cameras and hardware structure. The future cost could be easily reduced based on the new cameras introduced in the market. The new proposed device can couple measuring the instantaneous urological parameters and voiding visual inspection [25].

We speculate that there is an added value in a video record of the voiding process, especially in urinary incontinence. VVD is a more dignified way of assessing incontinence. In addition, many voiding behaviors and artefacts would be obvious on a video record. It’s not easy to assess this added value of video record, however being able to accurately measure and record urine volumes less than 50 ml, an objective method of quantifying the amount of leaked urine during coughing or straining in cases of stress urinary incontinence will be available at hand, no matter how small the leaked volume of urine was. Obviously this will be of value in both the initial diagnosis and assessment of severity of the condition, in order to select a suitable treatment modality as well as in follow-up to assess treatment outcome.
